# Innovative Approaches to Tin Recovery from Low-Grade Secondary Resources: A Focus on (Bio)hydrometallurgical and Solvometallurgical Methods

**DOI:** 10.3390/ma18040819

**Published:** 2025-02-13

**Authors:** Ewa Rudnik

**Affiliations:** Faculty on Non-Ferrous Metals, AGH University of Krakow, Al. Mickiewicza 30, 30-059 Krakow, Poland; erudnik@agh.edu.pl

**Keywords:** tin, leaching, recovery, solders, tin oxide, PCB, LCD, ITO

## Abstract

Tin, although not considered a critical material in all world regions, is a key material for modern technologies. The projected scarcity of tin in the coming decades emphasizes the need for efficient recycling methods to maintain uninterrupted supply chains. This review article focuses on the recovery of tin from low-grade secondary sources, specifically obsolete printed circuit boards (PCBs) and liquid crystal displays (LCDs). In both types of waste, tin occurs in various concentrations and in different chemical forms—a few percent as metal or alloy in PCBs and several hundred ppm as tin(IV) oxide in LCDs. This article presents pretreatment methods to preconcentrate tin and enhance subsequent leaching. It discusses not only conventional acid and alkaline leaching techniques but also the use of complexing agents and the challenges associated with bioleaching. Due to the dilution of the resulting leachates, advanced methods for tin ion separation and preconcentration before final product recovery are shown. Solvometallurgical methods employing deep eutectic solvents or ionic liquids, are also discussed; although promising, they still remain under development.

## 1. Introduction

**Figure 1:**
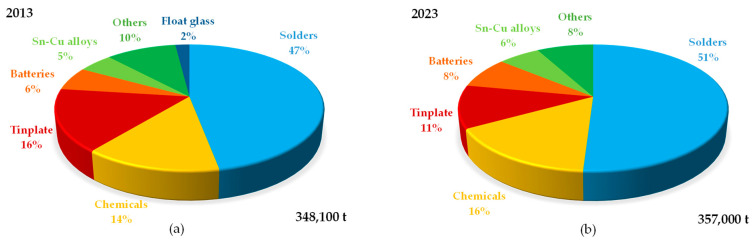
Changes in global tin demand by usage over decade: (**a**) 2013, (**b**) 2023 [20].

Tin is one of the earliest metals known to humanity, with a long history of mining and use [1], and remains vital in various practical applications today. Due to its ability to harden copper, tin was used in bronze artifacts as early as 3500 B.C., although its use as a pure metal began around 600 B.C. Throughout the ages, tin became increasingly important for producing alloys widely used for tableware, decorative items, and tinned iron vessels [2]. In the 19th and 20th centuries, its role expanded with the advent of tin plating, which revolutionized food preservation through the production of cans [3,4]. Currently, tin is an essential material in modern technology, especially in the electronics industry, where it is used in soldering due to its excellent conductivity and low melting point, making it a widely adopted replacement for lead [5,6]. Consequently, this sector accounts for about half of the total global demand for tin applications (Figure 1). Tin compounds are exploited also in high-tech applications, such as alkali-ion batteries [7,8,9], wires for superconducting magnets [10,11], materials for energy storage [12], and photovoltaics [13,14], contributing to renewable energy solutions.

Since tinplate packaging has increasingly been replaced by aluminum and plastic, and lithium-ion batteries—representing a significant emerging market for tin—are still under development, tin is classified as a non-critical metal in some countries, such as Australia, India, Japan, South Africa, and the European Union [15,16]. However, while the EU’s criticality matrix places tin just outside the critical zone due to its relatively low supply risk and projected decreasing trend in economic importance [17], tin is considered a critical metal in other regions, including Brazil, Canada, China, Indonesia, the United Kingdom, and the USA [15]. Notably, Bradley et al. [18] recently strongly recommended classifying tin as a critical metal for the EU, highlighting the significant risk of supply disruptions and emphasizing existing discrepancies in the definitions and values of criticality indicators. Moreover, modeling of global tin demand predicts continuous growth until 2080, with a noticeable decline only after 2200, driven by a significant reduction in metal supply [19]. It should be noted that a soft scarcity of tin is expected to emerge around 2050, when demand will surpass supply, leading to higher prices. It is forecasted that this will be followed by a decrease in demand due to rising prices, and by around 2150, it is probable that it will transition into a hard scarcity, where the required amounts of tin will no longer be deliverable.

Tin is a relatively scarce element, with an abundance in the Earth’s crust of approximately 2 ppm [21], ranking it 49th among naturally occurring elements. The primary tin mineral of commercial significance is cassiterite SnO_2_, with typical 0.4–1.5% tin concentration in ore [22,23,24], though smaller quantities of tin are also recovered from complex sulfides such as stannite Cu_2_FeSnS_4_, kesterite Cu_2_(Zn,Fe)SnS_4_, and canfieldite Ag_8_SnS_6_ [22]. The principal tin ore deposits are distributed irregularly along a “tin belt” encircling the Pacific Ocean [25]. This belt includes countries in Southeast Asia and stretches from Myanmar, Thailand, Malaysia, Sumatra, and West Kalimanan. Thus, it is evident that Myanmar and Indonesia together accounted for almost 37% (106,000 t) of global tin mine production in 2023 [26]. China was the leading tin producer in 2023, accounting for 23% (68,000 t) of the world’s output, while Peru ranked as the fourth-largest producer, contributing approximately 8% (23,000 t) [26].

**Figure 2:**
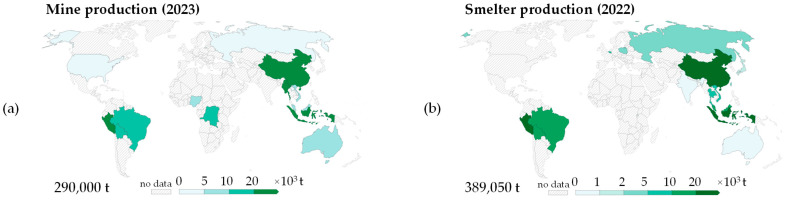
Global tin production by country: (**a**) mining in 2023, (**b**) smelting in 2022 [26].

The geographical distribution of tin reserves is limited to a few locations, leading to a high concentration of both mine production and refined tin production in a small number of countries, primarily located in Asia and South America (Figure 2). An expected 2.5-fold increase in global tin consumption by 2050 [27] can put pressure on tin supplies, particularly regarding long-term availability. This challenge involves ensuring resource security [28], improving production efficiency with environment protection, maintaining stable supply chains, changes in international trade flows, and pricing mechanisms [29] with restriction of illegal mining [30].

On the other hand, it is important to emphasize that the consumption of tin products results in a growing accumulation of tin-bearing waste and scrap. This highlights the need for the effective utilization of secondary materials to enhance a country’s ability to manage its tin resources, alleviate pressure on tin mines, and secure a durable and sustainable supply of the metal. It is especially important as recoverable tin from secondary resources (40,100,000 t Sn) is twice the amount of primary deposits (20,250,000 t Sn) [19], while according to the International Tin Association, only about 30% of refined tin produced in 2020 came from recycled sources such as used bearings, solder alloys, bronzes, and tinplate [20]. In fact, recent assessments [18] indicate that tin recycling indicators are lower (end-of-life recycling rate of 16%; end-of-life recycling input rate of 11%) than previously estimated (end-of-life recycling rate of 20–75%; end-of-life recycling input rate of 11–32%). Thus, only about 58% of processed tin is utilized currently, while the rest is either lost (28%) or remains within the new scrap cycle (14%).

Recycling of tin is primarily conducted through smelting. However, for multicomponent materials with low tin concentrations, energy-efficient alternative technologies show promise and align with the principles of the circular economy [31,32,33,34]. Therefore, the aim of this review is to compare (bio)hydrometallurgical and solvometallurgical methods for tin recovery from various waste materials derived from end-of-life electronics, focusing on the different chemical forms of tin and its concentration levels in printed circuit board scraps and discarded liquid crystal display panels.

## 2. Materials and Methods

The literature research was conducted between November 2024 and January 2025 using two major databases (Web of Science and Scopus), publisher-specific databases (American Chemical Society, IOP Science, MDPI, Royal Society of Chemistry, Taylor&FrancisOnline, ScienceDirect, SpringerLink, Wiley Online Library), and relevant online sources, focusing on keywords related to the topic. Among the identified references, 195 were selected as the most relevant for this review. These include scientific papers, books, and statistical databases. A systematic review of the data was achieved by cross-checking citations and analyzing supplementary materials attached to the original papers. Special focus was placed on research methodologies, significant findings, and the importance of experimental studies to the current research context, giving a base for detailed analysis and discussion.

## 3. Hydrometallurgy Versus Solvometallurgy

Hydrometallurgical and solvometallurgical methods involve the extraction and separation of metals using liquid solvents at moderate operating temperatures, commonly below 100 °C. Hydrometallurgy efficiently utilizes aqueous solutions of acids (e.g., H_2_SO_4_, HCl, HNO_3_), bases (e.g., NaOH, KOH, NH_3aq_), salts (e.g., NaCN, Na_2_CO_3_, NaCl, FeCl_3_), or chelating agents (e.g., EDTA, citric acid) for the leaching and selective recovery of metals from a variety of raw materials, including ores, concentrates, multicomponent wastes, or mine tailings, containing low percentages of target metals, sometimes as low as 1% [35]. Alternatively, biohydrometallurgical approaches have been developed to employ microbiologically assisted (bacteria, fungi) aqueous extractive metallurgy for natural sources, recycled materials, or residual waste, transforming uneconomic resources into valuable reserves [34,36]. This is also achieved through the application of methods that enable the concentration of metal ions from diluted solutions, such as solvent extraction, ionic exchange, or membrane processes. These techniques support the design of processes aligned with the principles of a circular economy, featuring closed flowsheets that minimize chemical consumption, regenerate reagents, generate minimal waste, and close water loops, thereby maximizing resource efficiency and sustainability [31].

Although processes based on aqueous solutions have been successfully used for almost 150 years [37], alternative non-aqueous solvents, first introduced at the turn of the 1940s and 1950s, have been increasingly promoted in extractive metallurgy in the 21st century [32,33,38,39]. Solvometallurgy (ionometallurgy) [33,40,41] employs ionic liquids and deep eutectic solvents for leaching and metal production, although in some cases, solvent–water mixtures with water content below 50 vol% can be used. Ionic liquids are salts composed of sterically demanding large organic cations (e.g., imidazolium, pyridazinium, thiazolium) and small organic (e.g., bistrifluoromethanesulfonimide) or inorganic (e.g., BF_4_^−^, HSO_4_^−^) anions [39]. By varying the cations or anions, the properties of ionic liquids can be tailored to specific applications. However, this customization often requires elaborate synthesis methods, making ionic liquids relatively expensive. Ionic liquids exhibit a wide liquid range, with melting points below 100 °C, excellent dissolution properties for many substances, and chemical and thermal stability. They are also conductive (up to 10 mS/cm) and can function as electrolytes with a wide electrochemical window, often exceeding 4 V [38]. In contrast, deep eutectic solvents are eutectic mixtures of two or more molecular compounds with a melting point lower than that of their individual components [40]. Most deep eutectic solvents consist of choline chloride combined with a hydrogen-bond donor (e.g., urea, ethylene glycol, malonic acid) or a hydrated metal salt (e.g., chloride) [33]. While deep eutectic solvents share similar properties with ionic liquids, their much simpler preparation makes them significantly easier and cheaper to produce.

**Figure 3:**
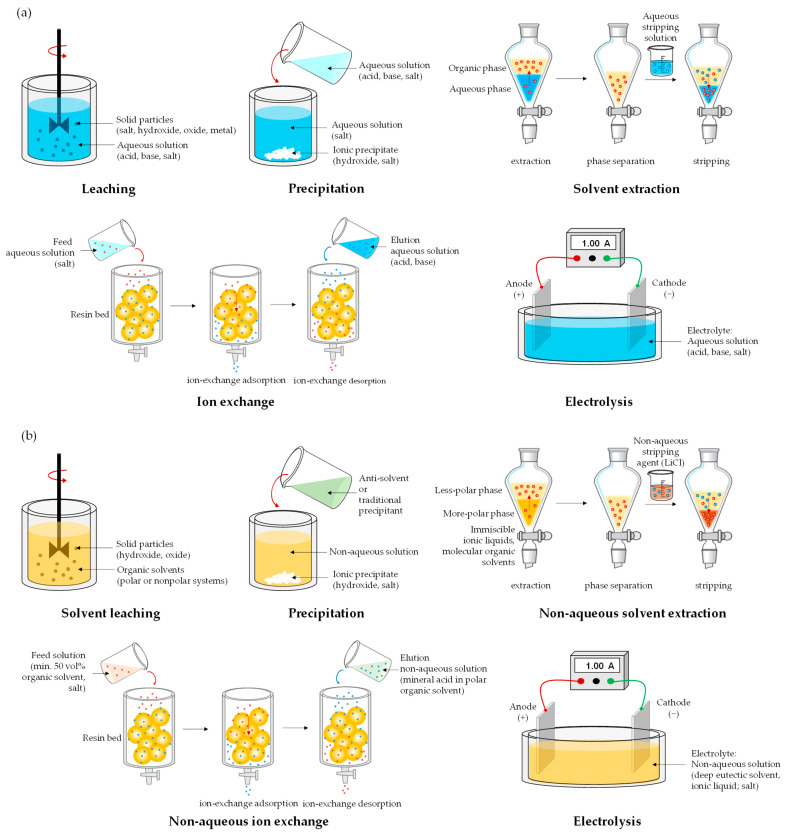
Comparison of hydrometallurgical (**a**) and solvometallurgical (**b**) unit operations.

The application of water-free systems in metal extraction from ores or waste materials offers selective leaching, excludes toxic leachants, eliminates extensive reagent consumption for barren material dissolution, reduces silica gel formation, decreases water consumption, improves energy efficiency through lower processing temperatures, facilitates the recovery of reactive metals, and supports integration with bioleaching. Since non-aqueous solvents are non-flammable, non-volatile, and biodegradable, they are considered safe and environmentally friendly [32,33]. Despite the clear benefits of using non-aqueous solvents, there have been no commercial breakthroughs in this branch of extractive metallurgy [38,40,41]. Key challenges include [41], among others, the high viscosity of non-aqueous liquids, limited long-term chemical stability under real operational conditions, recycling and reuse difficulties, and high costs for large-scale operations. However, it seems unlikely that innovations in hydrometallurgy based on non-aqueous solvents will emerge in the future, driven by groundbreaking scientific research that explores the potential of new systems, including in the areas of metal recovery and waste processing. This is particularly true since most unit operations are similar in both hydrometallurgical and solvometallurgical routes, with the primary differences arising from the use of different types of solvents (Figure 3).

Regardless of whether (bio)hydrometallurgical or solvometallurgical approaches are used, global efforts in scientific and industrial innovations in metal production and recovery are currently driven by the implementation of recycling schemes, with the ultimate goal of achieving complete circularity and conserving valuable resources [31,36,40]. These efforts also focus on the development of energy-efficient technologies for metal extraction and the treatment of end-of-life products and waste as new resources that require effective recycling strategies. In this context, the recovery of tin from various secondary sources, such as electronic waste and scrap metal, has gained significant attention.

## 4. Tin Recovery from Waste Printed Circuit Boards

### 4.1. Tin in Printed Circuit Boards

Printed circuit boards PCBs are among the most essential and valuable components of all electronic and electrical equipment. They are estimated to account for an average of 3–7% of the total e-waste stream [42,43,44,45], although their contribution can range from less than 2% to over 20%, depending on the type of discarded device [45,46]. Waste PCBs are multicomponent materials composed of metals (30–40%), polymers (20–30%), and ceramics with glass (30–50%), with these proportions varying based on the PCB type, manufacturer, and production year [47,48]. They contain a mixture of various elements, with copper (6–40%), iron (1–8%), aluminum (up to 7%), tin (1–6%), lead (1–4%), nickel (up to 5%), and zinc (up to 4%) being the primary metallic components [43,44,45,46,47,48,49,50,51], collectively representing over 95% of the metals in the waste [49]. Considering current metal prices (29.5 USD/kg for Sn, 15.1 USD/kg for Ni, 8.8 USD/kg for Cu, 2.8 USD/kg for Zn, 2.5 USD/kg for Al, and 1.9 USD/kg for Pb [52]), tin holds a high economic value [53]. Tin ranks second to fourth, alongside copper and silver, among the primary metals to be recovered from waste PCBs [54], although precious metals are typically identified as the main contributors to profitability [48].

Tin in PCBs (Table 1) is primarily used as a protective finish on copper tracks for enhanced durability, better conductivity, and solderability, as well as in solders to establish connections between components and tracks on the board [55,56]. Leaded solders were historically prevalent, particularly the eutectic 63%Sn-37%Pb alloy, valued for its low melting point (183 °C), excellent wettability, high conductivity, and low cost [56,57]. However, the Restriction of Hazardous Substances (RoHS) Directive, implemented in the EU in 2006 [58], restricted the use of leaded solders in electric and electronic products due to the proven harmful effects of lead and its significant environmental risks in e-waste [59,60,61]. Consequently, tin-based solders containing bismuth, zinc, silver, and/or copper have now replaced the traditional Sn-Pb alloy [56,57]. Instead, the average tin content in waste PCBs produced over the last forty years has remained relatively stable, at about 4% [44,49,62].

The detailed concentration of tin in waste PCBs depends on factors such as the type of device [43,54,65,66], sampling [49,65], particle size fraction [44,49,51,54], and the chemical analysis procedure used [47,51,67,68]. For example, Touze et al. [49] reported an approximately twofold enrichment of tin, with the coarse fraction (particles > 500 μm) containing 1.1% Sn, compared to 0.6% Sn in the fine-grained fraction (particles < 250 μm) of ground PCBs. In contrast, Anić-Vučinić et al. [44] observed that tin content was lowest in the largest grain size fraction (1–4 mm) and highest in the small particle fraction (0.1–0.5 mm). However, when bare base plates were analyzed, tin was found to accumulate predominantly in the largest particle fraction (1–4 mm). Different trends of tin accumulation in fractions of shredded PCBs [44,49] indicate that its concentration measurements are characterized by high uncertainty due to the significant variability in sampling.

Notably, the digestion methods used for PCB sample preparation prior to solution analysis significantly affect the detected metal contents. Tunali et al. [67] compared three methods (U.S. EPA Method 3051A, Modified Microwave Digestion Method, and U.S. EPA Method 3050B) for determining metal contents in waste PCBs from various discarded devices (mobile phones, smartphones, and laptops). They found that higher temperatures, longer digestion durations, and the use of more chemical reagents could enhance metal dissolution efficiency for most elements. Therefore, the choice of preparation protocol should be tailored to the type of metal being analyzed. Similarly, Das and Ting [68] observed discrepancies in tin concentrations (from 3.2% to 6.7% Sn) in the same waste PCBs, depending on the dissolution method used. Their study compared eleven methods, including standardized protocols (U.S. EPA Method 3050B; ASTM D6357-11), microwave-assisted and ultrasound-assisted digestions, as well as variations in the acid mixture formulations used, demonstrating that those based on concentrated hydrochloric acid HCl were the least efficient. In turn, Van Yken et al. [51] evaluated smelting and ashing as potential pre-treatment methods prior to analytical characterization. Smelting was found to reduce the accuracy of subsequent analysis due to the volatilization of certain metal species at high temperatures. In contrast, ashing proved to be a viable pre-treatment method. Among the four analytical methods tested, microwave-assisted multi-acid digestion demonstrated the highest precision and accuracy. Regardless of the pre-treatment and digestion approaches, the choice of analytical method—typically inductively coupled plasma mass spectrometry ICP-MS, inductively coupled plasma optical emission spectrometry ICP-OES, or atomic absorption spectrometry AAS—also imposes detection limits that influence the quantification of specific elements. These studies revealed that the selection of analytical procedure significantly impacts the accuracy of observed metal contents in PCBs, underscoring the importance of standardizing methodologies and utilizing certified reference materials.

### 4.2. Physical Pre-Treatment

The complex composition and heterogeneous structure of waste PCBs necessitate pre-treatment before metal extraction steps [69]. This process may include mechanical and/or thermal dismantling, optional size reduction (shredding, crushing, grinding), and upgrading separation techniques based on differences in particle shape, size, density, and wettability as well as magnetic or electrical properties [70,71,72]. Chemical methods are also employed, primarily for dissolving solder masks, solders, and resins, as well as for organic swelling and depolymerization [48].

Tin in PCBs predominantly exists as thin metallic coatings on copper tracks and in solders [55,56]. Thus, its behavior during pre-treatment stages depends on the physical and chemical properties of the pure metal and its alloys. Tin and its alloys are relatively soft, malleable, have low melting points, medium density, and weakly paramagnetic properties (Table 2). As a result, they melt by heat treatment, show minimal interaction with magnets during magnetic separation, and can be concentrated by gravity or electric field.

Desoldering waste PCBs is the first, and a key step, in successful recycling processes. It enables the dismantling of electronic components from the board, solder regeneration, and the recovery of pure tin. This step can be performed through abrasion of solder joints using a grinder, melting the solder with various heating methods, or dissolving it with chemical reagents [75,76]. Mechanical grinding is suitable for processing individual boards; however, it is challenging to apply uniform grinding techniques due to the structural variability of different PCBs, which limits its industrial applicability. Heat treatment allows for the simultaneous processing of large quantities of waste PCBs. However, differences in heat capacities and the uneven distribution of components can lead to inconsistent temperature increases in different regions of the board. This can damage electronic components that might otherwise be reusable. The temperature required for desoldering closely correlates with the melting points of solder alloys. While raising the temperature above the solder’s melting point accelerates desoldering, temperatures above 250 °C do not further increase the melting rate. Furthermore, temperatures exceeding 280 °C result in the emission of toxic gasses [76]. Additionally, since tin-based solder materials melt at lower temperatures than copper (1083 °C), their components can diffuse into it, forming metallic compounds that lower copper’s melting point and affect the quality of the recovered metal. To address this, and to recover solder materials in a homogeneous form without evaporating metals such as lead, tin, and zinc, Hossain et al. [77] developed a fast heating process in a reducing atmosphere. This process recovered a tin-based alloy (86% Sn-8.8% Zn-3.1% Pb-1.8% Cu) at 500 °C within 5–10 min from waste PCBs containing 24.6% Cu, 4.4% Sn, 1.3% Zn, 0.4% Pb, 2% Al, and 3.3% Fe. The recovered alloy demonstrated an ultimate tensile strength about 30% higher than that of a standard Sn-9% Zn solder alloy.

Based on the differences in melting points, Meng et al. [78] used a supergravity technique to separate and recover approximately 50% of tin and lead as an alloy (Pb/Sn 0.66) from waste PCBs containing 52% Cu, 10% Sn, 7% Zn, 5% Pb, 7% Zn, and 1% Al. The process was carried out at 410 °C for 5 min with a gravity coefficient of 1000, generated by a centrifugal apparatus. Under these conditions, differences in the melting points (or densities) of solid particles and liquid melts caused the particles to be distributed and separated gradually along the centrifugal direction. However, tin formed solid solutions with copper and silver during the process, which reduced its recovery rate. The recovered Pb-Sn alloy was suitable for industrial applications, such as solder production and ornamental manufacturing.

**Figure 4:**
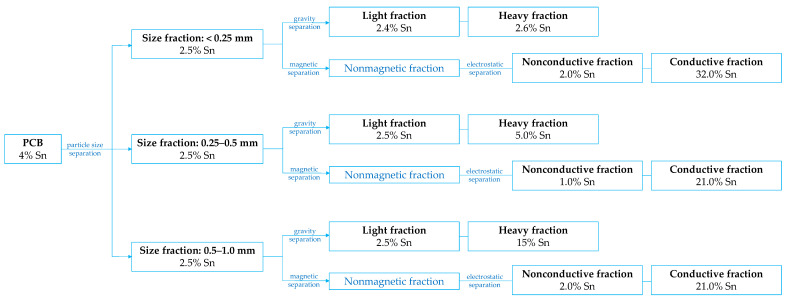
Comparative diagram of different separation methods for the enrichment of tin in milled waste PCB, based on ref. [79,80].

Veit et al. [79,80] applied a combination of mechanical processes to obtain particle fractions with higher metal concentrations. They used PCB scraps containing 4% Sn, which were sequentially divided into three particle size fractions (ranging from below 0.25 mm to up to 1 mm), with each fraction subjected to density or magnetic followed by electrostatic separation (Figure 4). It was found that the concentration of tin (2.5%) was uniform across different particle fractions. However, its percentage significantly increased to 15% in the heavy (sink) fraction during gravity separation in tetrabromoethane (density 2.5 g/cm^3^) of the larger particles (0.5–1 mm) [79]. Additionally, tin content increased several times (to 21% or 32% Sn) in the conductive fractions across all particle sizes during electrostatic separation of nonmagnetic parts [80].

Chao et al. [81] conducted a detailed size fractionation of crushed PCBs (3.4% Sn), sieving the material into seven particle size portions ranging from below 0.074 mm to above 1.25 mm. Tin was predominantly concentrated in the larger particle size ranges, particularly in three fractions with diameters of 0.3–1.25 mm (4.4–4.8% Sn) and one fraction of 0.15–0.3 mm (3.2% Sn). Notably, these fractions accounted for approximately 65% of the total mass. Electrostatic separation of each particle fraction further enriched the conducting fraction, achieving tin concentrations of up to 7.9% Sn (0.9–1.25 mm) and even 9.6–10.7% Sn (0.15–0.9 mm). In contrast, the nonconductive parts typically contained less than 0.1% Sn, except for the 0.3–0.6 mm fraction, which contained 0.3% Sn.

Although many studies [44,49,81,82] report that tin predominantly concentrates in coarse particle fractions (typically 0.5–1 mm) of crushed PCBs, some data [44,83] indicate an opposite trend, highlighting the influence of both the raw material’s origin and the analytical method employed. For instance, Barnwal and Dhawan [83] investigated tin concentrations in various size fractions of discarded PCBs from laptops and desktop computers (4.9% Sn). Their XRF analysis revealed the highest tin concentration (7.6%) in particles sized 0.1–0.2 mm, while only 2.2% Sn was identified in the 0.5–1 mm fraction.

**Figure 5:**
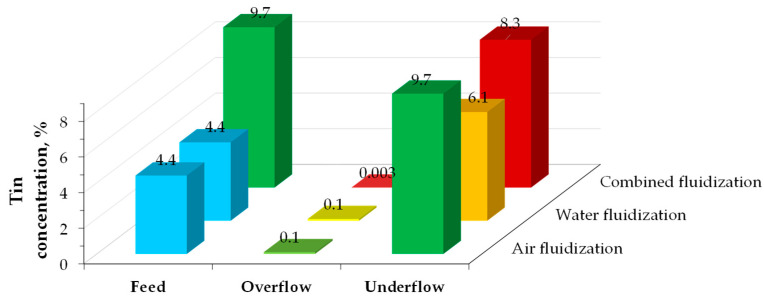
Tin concentration in feed material (grinded discarded PCBs) and different fractions generated in fluidization separation processes, based on ref. [83].

The crushed discarded PCBs (4.4% Sn) underwent separation via fluidization processes in air, water, and combined routes [83]. In this technique, separation into heavy and light fractions occurs in a moving fluid; smaller or lower-density particles are carried away with the medium (overflow fraction), while larger or higher-density particles remain (underflow fraction). The combined fluidization approach—using the underflow fraction from air fluidization as feed for water fluidization—achieved an 80% separation efficiency, with the underflow fraction consisting of 87% metals (Al, Cu, Zn, Pb, Sn). However, from the perspective of tin enrichment, air fluidization proved to be the most effective process as its content more than doubled (Figure 5).

Waste printed circuit boards, composed of diverse materials, allow the use of differences in density and wettability to separate polymers from metals. Flotation, reverse flotation, and froth flotation processes utilize fine particles suspended in an aqueous medium [71]. These methods divide particles into floating non-wettable (hydrophobic) polymers (flotation) or metals (reverse flotation), while wettable (hydrophilic) particles settle at the bottom. In froth flotation, selective air bubble adsorption enhances the separation of targeted particles. The efficiency of separation is directly proportional to the differences in surface properties among the particles, which can be further improved by adding chemical reagents. Collectors are used to enhance the hydrophobicity of the desired particles through adsorption on their surfaces, while frothers stabilize the foam and disperse air, thereby accelerating the flotation process.

Franke et al. [84] investigated flotation for the separation of ground PCBs (particle sizes ranging from below 0.09 mm to 1.4 mm). They tested three additives—tannic acid, dimethoxy dipropylene glycol, and 2-octanol—in various combinations, identifying ether (157 mg/L, 200 L/h air, 50 g/L PCBs) as the most effective for metal recovery. However, the purity of the recovered metals was lower compared to other methods, such as gravity or electrostatic separation, applied to the same feed material. The hydrophilic product (metals) contained 7.8% Sn with an 84% recovery rate, while the hydrophobic product contained only 1.1% Sn. In turn, Barnwal et al. [85] utilized a finer PCB powder (below 0.2 mm) for flotation in tap water without additional reagents. They observed an increase in the metallic fraction from 14% in the feed material to 92% in the concentrate, which consisted mainly of copper (78%) along with 8.3% Sn and 5.5% Pb. As tin losses occur during flotation into the polymer fraction, Das et al. [86] conducted a series of froth flotation experiments to reduce the collection of metals in hydrophobic plastic particles. They tested setups with no additives, with a collector, with a frother, and with both compounds (unspecified). The transition of metals, including tin, into non-froth products (metallic phase) decreased over time in all cases, but the best results were achieved without surfactants. The addition of surfactants reduced tin recovery from 92% (no additives) to 65% (with both additives). Consequently, depending on the frother dosage (0–0.15 mg/kg) the tin content in the metallic phase increased in a range of 7.4–8.2%, compared to 4.8–5.7% in the froth product and 6.8% in the initial feed material.

### 4.3. Hydrometallurgical Treatment

Tin is a non-noble metal (E°_Sn/Sn(II)_ = −0.136 V) with amphoteric properties [87]. It reacts with strong acids and bases, while showing relative resistance to weak acids and bases. Tin readily dissolves in concentrated solutions of hydrochloric and sulfuric acids, or sodium hydroxide, forming soluble Sn(II) or Sn(IV) compounds, respectively [88]:

Sn + 2HCl →  SnCl_2_ + H_2_ (1)

Sn + 2H_2_SO_4_ →  SnSO_4_ + SO_2_ + 2H_2_O (2)

Sn + 2NaOH + 4H_2_O →  Na_2_[Sn(OH)_6_] + 2H_2_ (3)

Dilute nitric acid is said to slowly produce soluble tin(II) nitrate, although the presence of bivalent metal ion can be questionable [87]:

4Sn + 10HNO_3_ →  4Sn(NO_3_)_2_ + NH_4_NO_3_ + 3H_2_O (4)

In contrast, concentrated nitric acid oxidizes metallic tin to Sn(IV), which precipitates as hydrated oxide SnO_2_∙H_2_O (also stannic acid H_2_SnO_3_) [88]:

Sn + 4HNO_3_ →  SnO_2_↓ + 4NO_2_ + 2H_2_O (5)

Several Sn(II) salts are water-soluble, but are susceptible to hydrolysis, leading to the formation of hydroxo salts or hydrous tin(II) oxide, e.g.,

SnCl_2_ + H_2_O →  Sn(OH)Cl↓ + HCl (6)

SnSO_4_ + H_2_O →  SnO↓ + H_2_SO_4_ (7)

Sn(II) ions in solution are also prone to oxidation to Sn(IV) by atmospheric oxygen:

SnCl_2_ + ½O_2_ + 2HCl →  SnCl_4_ + H_2_O (8)

which is followed by the formation of soluble complexes in the presence of excess halide ions or acid:

SnCl_4_ + 2HCl →  H_2_SnCl_6_ (9)

Thus, the complex chemistry of tin and its compounds in aqueous solutions picks the leaching conditions for waste PCBs, the type of products generated, and the efficiency of metal recovery (Table 3).

Ranitović et al. [89] compared the leachability of tin and lead from mechanically treated waste PCBs (3.3% Sn, 2.5% Pb) using HCl (2–6 M), HNO_3_ (1–3 M), and NaOH (0.5–5 M; m–nitrobenzoic acid as oxidizer) solutions under varying leaching times (1–6 h) and temperatures (60–90 °C). The efficiency of tin leaching (24–92%) increased with HCl concentration and time, with temperature being the dominant enhancing factor. However, secondary precipitation of PbCl_2_ (along with AgCl) posed a significant challenge, reducing the lead dissolution rate (14–42%). HNO_3_ solutions proved to be the most selective lixiviant, dissolving over 98% of lead but only 10–16% of tin. Interestingly, tin leaching efficiency decreased with increasing acid concentration, temperature, and process duration, likely due to the precipitation of SnO_2_·H_2_O, which hinders straightforward leaching and subsequent recovery in hydrometallurgical steps. NaOH solutions showed limited selectivity for leaching both metals, with tin recovery rates (32–62%) increasing with temperature and base concentration but decreasing over time. This behavior was attributed to simultaneous dissolution and precipitation of metal hydroxides, which contained about 50% of tin, highlighting a significant drawback of the NaOH lixiviant.

**Figure 6:**
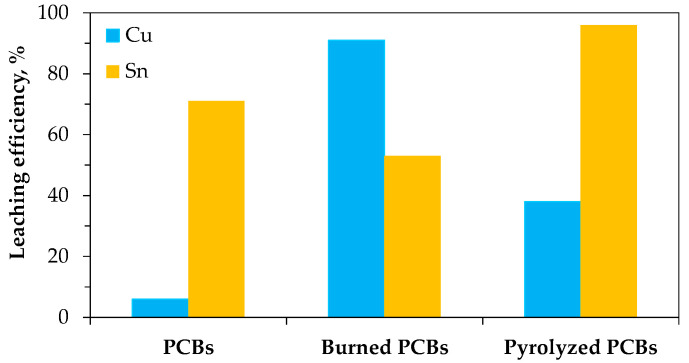
Effect thermal treatment (900 °C) of PCBs on leaching efficiency of tin and copper (1 M HCl, 80 °C, 3 h), based on ref. [112].

Effective tin-leaching behavior in HCl [90,91,93,94,99,100], alongside the poor dissolution observed in HNO_3_ [90,95,108,109,110] solutions, has been further corroborated by numerous studies, highlighting their potential as selective leachants for separating tin from copper [94,100] or lead [90,108,109,110]. Of note is the work by Ilyas et al. [111], which reported 30–90% dissolution of Sn-Pb solder in 4 M nitric acid at 30–90 °C (3 h), followed by secondary tin oxide precipitation upon cooling. The acid leachability of metals can be further enhanced by pretreatment of the waste through swelling in organic solvents (e.g., DMF) [90], burning, or pyrolysis [112]. In the latter cases, the behavior of tin and copper differs since depending on the air supply (burning involves air access, while pyrolysis does not), both metals transform into oxides with varying reactivity and solubility in HCl solution (Figure 6).

The rate and selectivity of metal leaching with HCl can be adjusted by altering the ionic chemistry of the solution. Mixtures of basic mineral acids with HCl have been investigated, with results consistently highlighting it as a key component for enhancing tin recovery rates convincing selectivity against copper [93]. Zhao et al. [100] employed electro-generated chlorine (produced at the anode), which dissolves in the acid solution to regenerate HCl and form additional leaching agents such as HClO and Cl_3_^−^. This approach improved tin dissolution by 25%, reaching nearly 100%, but significantly reduced the process’s selectivity due to the formation of soluble metal chloride complexes. Conversely, Jung et al. [101] and Kim et al. [113] added tin(IV) chloride to the acid to enhance solder dissolution through a synproportionation reaction:

Sn + SnCl_4_ →  2SnCl_2_ (10)

They reported up to 99% tin dissolution, demonstrating the effectiveness of this method for PCB dismantling [101]. An intriguing phenomenon was observed during the treatment of Sn-Cu-Ag solder. Initially, copper and silver dissolved under the action of Sn(IV) ions. However, at a later stage, copper ions were cemented by metallic tin, which in turn enhanced the leachability of tin [113]:

Cu + SnCl_4_ →  CuCl_2_ + SnCl_2_ (11)

Sn + CuCl_2_ →  SnCl_2_ + Cu (12)

Although sulfuric acid H_2_SO_4_ is a commonly used, inexpensive leaching agent for various raw materials, it has proven ineffective for tin recovery from waste PCBs and solders, with typical tin leaching efficiencies of about 2% [90,93,96,108,114]. In certain cases [96,97], tin leaching rates reach up to 20–25% when conducted at elevated temperatures. However, Lisińska et al. [98] showed significantly higher tin leaching rates at temperatures of 40–80 °C and extended process duration (8 h), with much lower efficiencies in 5 M H_2_SO_4_ (64–69%) compared to 2 M solutions (79–100%). The dissolution of tin was accompanied by the transfer of certain ions, such as Fe^3+^ (98%), Zn^2+^ (50%), and Ni^2+^ (20%), into the solution, while copper and lead remained in the solid residue. The presence of hydrogen peroxide H_2_O_2_ can also help in the complete dissolution of the metal [115]. Indeed, not aerated diluted sulfuric acid has shown good potential to selectively dissolve aluminum, iron, and zinc, leaving tin and copper. Guo et al. [96] utilized this effect to develop a two-step leaching process. In the first step, tin was selectively leached using an H_2_SO_4_−CuSO_4_ solution, where a displacement reaction between Cu^2+^ ions and metallic tin resulted in the accumulation of SnSO_4_ in the aqueous phase while copper remained in the solid residues. The second step involved the recovery of hydrolytic precipitates of SnO_2_ under oxidative conditions.

Cui and Anderson [50] investigated the addition of inorganic acids to a NaBr-Br_2_ system. Bromine reacts with water to form hydrobromic acid HBr and hypobromous acid HBrO, both of which exhibit a high oxidation-reduction potential. When the bromide-bromine mixture was enriched with HCl, HNO_3_, or H_2_SO_4_, it achieved high metal dissolution rates (90–99%), effectively leaching not only tin, zinc, and nickel but also copper, silver, gold, and palladium.

Zhang et al. [102] proposed a mixture of fluoroboric acid HBF_4_ and hydrogen peroxide H_2_O_2_ as a novel lixiviant for the selective removal of Sn-Pb solder from PCBs. They achieved complete recovery of the alloy, with minimal copper dissolution, within a short reaction time (0.5 h), using 3 ± 0.5 M acid and 0.5 ± 0.1 M oxidizing agent. The concentration of H_2_O_2_ was critical for solder dissolution, as higher dosages reduced the leaching rate. Excess peroxide promoted autogenous decomposition, generating oxygen, which facilitated the formation of SnO, subsequently oxidized to SnO_2_. This passivating layer prevented further metal dissolution and simultaneously increased copper leaching from PCBs, though only 6% of copper was dissolved at 0.4 M H_2_O_2_. Ping et al. [116] further investigated the HBF_4_−H_2_O_2_ system, finding a relationship between the desoldering reaction time and the acid-to-oxidant concentration ratio. The shortest reaction time (1 h) was achieved at a HBF_4_:H_2_O_2_ volume ratio of 1.7. Both lower (up to 0.8) and higher (up to 2.6) ratios prolonged the reaction duration. The dissolving mixture could be recycled by adding oxidant and, if necessary, acid, allowing reuse for solder removal without wastewater discharge. The point of regeneration could be identified by the oxidation-reduction potential of the solution, which changed consistently due to the action of Cu-Sn-Pb microcorrosion cells and precipitation of copper, corresponding to the dissolution of tin upon H_2_O_2_ addition. As in other leaching processes, tin oxides precipitated if the electrolyte’s acidity was insufficient. However, with additional acid, Sn(BF)_2_ and Sn(BF_4_)_4_ were formed in solution. Notably, other oxidizing agents, such as Ti^4+^ or Fe^3+^ ions, in HBF_4_ solutions were ineffective for tin dissolution from PCB solder, even with adjusted dosages, reaction temperatures, and times [102].

In contrast to inorganic acids, organic acids have been sporadically studied as potential lixiviants for tin recovery from waste PCBs and spent solders [103,104,114]. Acetic acid CH_3_COOH proved completely ineffective for tin dissolution, citric acid H_8_C_6_O_7_ dissolved only 18% of tin at room temperature [114], while oxalic acid H_2_C_2_O_4_ could extract about 20% of tin and even 93% in a mixture with H_2_O_2_ from pyrolyzed PCBs [104]. Zhang et al. [103] investigated methanesulfonic acid CH_4_SO_3_ with H_2_O_2_ as an additive, testing various leaching parameters (concentrations, reaction time, temperature). They identified optimal conditions for nearly complete tin dissolution using 3.5 M acid and 0.5 M oxidant concentrations at room temperature. This process enabled the effective dismantling of PCBs due to the complete dissolution of lead, while demonstrating selectivity against copper leachability (up to 7%).

Acidic solutions of ferric salts, such as chloride [117] or sulfate [118], have also been applied as inexpensive leaching agents due to their strong oxidative properties (E°_Fe(II)/Fe(III)_ = +0.77 V) which can oxidize most metals from PCBs. However, the efficiency for tin dissolution has not been reported, although it can be leached along with copper [117].

Acidic spent tin stripping solutions were used [119,120]. These are waste solutions originally from PCBs production which contain typical base metal ions and nitric acid. Several studies have shown that such solutions are able to total Sn-Pb alloy solder dissolution, although secondary stannous oxide precipitate due to gradual decreasing of concentration of hydrogen ions with time [119]. Tan et al. [118] noticed that 55% of tin extracted accumulated in solution, while the remaining 45% precipitated as SnO_2_ after leaching.

Alternatively to acid leaching, alkaline leaching can also be employed due to the amphoteric properties of tin. Yang et al. [105] investigated alkaline pressure leaching under oxidizing conditions, using NaOH as the lixiviant and oxygen gas as the oxidizing agent. They observed efficient dissolution of tin along with lead and aluminum, while copper remained in the solid residue. Subsequently, nearly all lead and zinc were precipitated from the solution with sodium sulfide Na_2_S, followed by electrowinning of high-purity tin (min. 99.8%). The process flowchart included reusing the spent electrolysis electrolyte in the leaching step.

**Figure 7:**
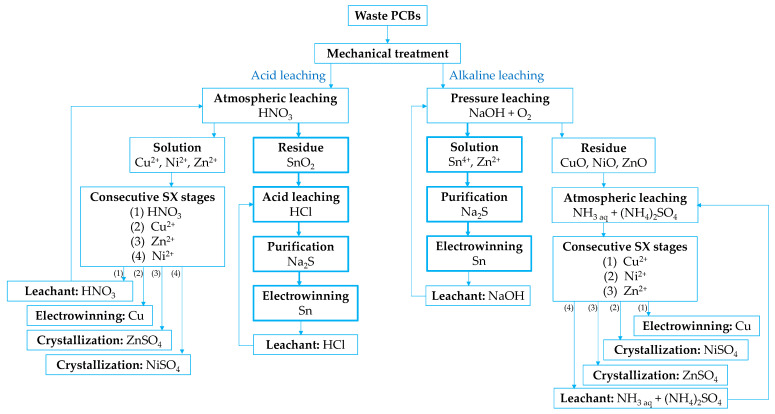
Schemes of metal recovery from waste PCBs, based on ref. [121]. SX—solvent extraction.

Nan et al. [121] conducted a comparative analysis of two metal leaching and recovery schemes from waste PCBs (Figure 7). The simulations revealed that while the acidic leaching process achieved higher recovery rates for base metals (92% for Sn) and offered advantages such as lower electricity consumption and reduced wastewater generation, it had a significantly higher environmental impact (12.5 t CO_2_ eq/t PCB) due to the chemical inputs required for the HNO_3_ leaching process. In contrast, the alkaline leaching process used fewer chemicals, showed comparable or slightly lower metals recovery (96% for Sn), generated less solid waste and fewer off-gasses, and resulted in a lower overall environmental footprint (11.4 t CO_2_ eq/t PCB). Thus, although the acidic leaching process can be more efficient for metal recovery, the alkaline leaching process demonstrates greater environmental sustainability and holds significant potential for further technical optimization.

Unconventional leaching agents have also been proposed for the chemical treatment of PCBs. These include complexing agents such as EDTA (sodium salt) [97], a mixture of sodium citrate H_5_C_6_O_7_Na_3_, ammonium phosphate (NH_4_)_3_PO_4_, and hydrogen peroxide H_2_O_2_ [122]. Abdo et al. [97] reported that tin extraction using EDTA was nearly complete compared to other agents like HNO_3_ (up to 30%) or H_2_SO_4_ (up to 18%) at elevated temperatures. However, the process was pH-dependent, with neutral conditions significantly hindering efficiency. The stannate ions formed during the process were precipitated with sodium hydroxide to produce spherical SnO_2_ nanoparticles (8–12 nm) suitable for photocatalytic degradation of methylene blue. The combination of citrate and phosphate salts under oxidizing conditions [122] was found to be selective for copper dissolution, leaving tin and other metals largely unaffected. Although tin was eventually transferred to the solution over time, its ion concentration did not exceed 0.8 g/L, even after extended leaching durations of (up to 6 h). This selective removal of copper facilitated the subsequent separation of other metals in more targeted and efficient ways like tin extraction (60%) with a combination of thiourea CS(NH_2_)_2_, H_2_O_2_ with oxalic acid C_2_H_2_O_4_ or enhanced gold recovery (at slowed down tin dissolution) if potassium thiocyanate KSCN was added to the mixture [123].

Electrochemical dissolution has also been employed for metal recovery. Tang et al. [106] utilized PCBs coated with tin as the anode during electrolysis in methanesulfonic acid. Under optimal conditions, approximately 85% of the tin was removed from the anodic material, leaving copper unaffected, and subsequently deposited on the cathode. Similarly, Zhang et al. [107] used NaOH as the electrolyte to extract solder from the PCB surface under various conditions, demonstrating that complete recovery could be achieved in 2–3 M NaOH within 2 h, using anodic current densities of 3–6 A/dm^2^ at 80–90 °C. In another study, Fogarasi et al. [124] applied electrochemical dissolution for the selective recovery of tin from waste solder generated in a secondary stream during copper recovery from waste PCBs (following copper dissolution in an FeCl_3_–HCl etchant). During electrolysis, tin accumulated in the H_2_SO_4_ electrolyte and was subsequently deposited on the cathode, while lead dissolved from the solder anode precipitated as sulfate.

Smelting of e-scraps produces polymetallic and multiphase high-copper alloys [125,126,127,128,129,130,131,132,133]. Thermal treatment enriched the material in tin (1.7–17%) by removing nonmetallic components and transforming the metal into new phases, mainly Cu-Sn solid solutions or intermetallic compounds [125,126,127,128,129,130,131], as well as Ag_3_Sn intermetallics [125,128,129,130]. Leaching [127,128,129,130,133] or anodic dissolution [125,126,127,128,130,132] conducted in acids (H_2_SO_4_, H_2_SO_4_ + NaCl, HCl) [125,130,132] or in ammoniacal solutions (chloride, sulfate, carbonate, thiosulfate systems) [126,127,128,129,130,133] distributed tin into different phases (slime, electrolyte, cathodic deposit), depending on the composition of the electrolyte. Ammoniacal leaching or anodic dissolution of the alloys accumulated tin in solid residues, mainly as unreacted intermetallic phases [126,127,129,130,133] from which tin can be completely leached using HCl [126]. In turn, during electrochemical dissolution in H_2_SO_4_, 60% of tin could be recovered on the cathode as a Cu-Sn alloy, with the recovery rate further enhanced to 85–90% by the addition of chloride ions to the electrolyte [125]. It is noteworthy that anodic dissolution of the alloy in H_2_SO_4_ occurred at almost constant potential, but the addition of chloride ions resulted in periodical inhibition of the process due to blocking of the anode surface by insoluble compounds, mainly PbCl_2_ and CuCl. Increase in both NaCl concentration and temperature enhanced dissolution of the secondary precipitates into chloride complexes and further dissolution of the alloy.

Guo et al. [134] proposed an efficient alkali-fusion-leaching-separation process for crushed PCBs. This method employed a mixture of sodium hydroxide and sodium nitrate NaOH-NaNO_3_ as a flux to convert amphoteric metals into water-soluble salts. Following water leaching, selective precipitations were carried out; copper was precipitated with glucose as copper(I) oxide Cu_2_O, tin with lime as calcium stannate CaSnO_3_, and zinc and lead with sodium sulfide as their respective sulfides ZnS and PbS. Under optimized conditions, the process achieved a tin recovery rate of approximately 91%.

The typical tin product recovered from waste PCBs is SnO_2_ [93,94,95,96,97]. It is either precipitated as a secondary product during the leaching stage under oxidative conditions [95] or formed in a subsequent step using an alkali [93,94,96,97]. SnO_2_ can be further dissolved to produce an acid chloride electrolyte for metallic tin electrowinning [95]. Alternatively, tin can be electrodeposited in pure form from the purified leachate [105], as tin ions remain in the aqueous phase and are not transferred into sulfides during precipitation reaction [105] or into the organic phase during solvent extraction [135]. Additionally, tin can be recovered as a Cu-Sn alloy during the direct electrolysis of the leachate [136]. In some cases, tin can also be recovered alongside lead by cementation, enabling the recovery of the solder alloy [91,92].

### 4.4. Biohydrometallurgical Treatment

Biohydrometallurgical treatment of waste PCBs involves the use of bacteria or fungi to transform metals into soluble compounds (bioleaching), binding from solution (biosorption) or active uptake of metals ions (bioaccumualtion) through the action of microorganisms [137,138,139]. Although this approach is an economically viable and ecological alternative for recovering metals like copper, gold, or silver and boasts a smaller carbon footprint (around 43% less) compared to traditional hydrometallurgical (chemical) methods [136], its applicability to metals like tin or lead remains limited [64,111,138,139,140,141,142,143,144,145]. Table 4 shows tin bioleaching data obtained under various conditions using bacterial or fungal cultures.

Acidophile moderately thermophilic bacteria like *At. ferrooxidans* or *At. thiooxidans* and *At. caldus* generate an acidic, oxidizing leaching environment through the oxidation of sulfur [111,140,143], sulfide minerals (e.g., pyrite) [141], or ferrous sulfate FeSO_4_ [64,145] to sulfuric acid and/or ferric ions in an aerated medium:

(13) $$2S+3O_{2}+2H_{2}O\;\overset{Bacteria}{\to }\;{{\mathrm{HSO}}_{4}}^{-}\;+\;{{\mathrm{SO}}_{4}}^{2-}\;+\;{3H}^{+}$$

(14) $$2{\mathrm{Fe}}^{2+}+½\;O_{2}+2H^{+}\;\overset{Bacteria}{\to }\;2{\mathrm{Fe}}^{3+}\;+\;H_{2}O$$

This is followed by the oxidizing action of ferric ions on metals, converting them into soluble sulfates:

2Fe^3+^ + M →  2Fe^3+^ + M^2+^ (15)

Bioleaching under such conditions partially transforms metallic tin into ions during the initial stages of the process [141,143,145] and/or at low PCB concentrations [143]. However, prolonging the leaching process or increasing the pulp density reduces the concentration of metal ions in the solution due to the precipitation of oxides (postulated as SnO but not experimentally verified) [64,140,143]. This results in the complete lack of tin recovery in soluble form in the leaching medium at the final stages [140,141,143,144].

**Figure 8:**
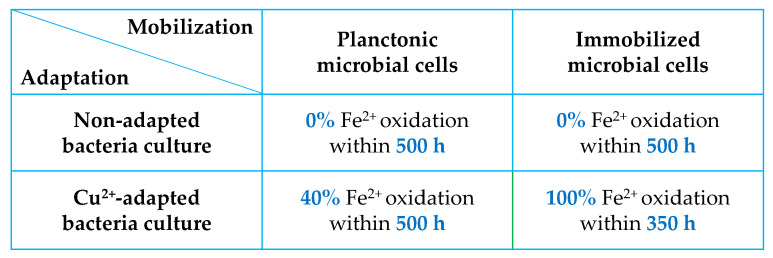
Microbial oxidation of 10 g/L Fe^2+^ ions by non-adapted and Cu^2+^-adapted mixed mesophilic bacterial cultures (*L. ferriphilum, Ap. cupricumulans, At. caldus*) in the presence of 10 g/L Sn^2+^ ions. Microbial cells planctonic (suspended in aqueous medium) or immobilized on polyurethane foam. Based on ref. [64].

Although PCB leachates may contain small amounts of tin ions, with some precipitated as tin oxide, these ions exhibit an inhibitory effect on microbial cultures, which is particularly important in a one-stage reactor setup. On the other hand, the precipitation of tin oxide mitigates the toxic effects of tin ions on microbial activity to some extent, despite the culture’s adaptation to metals [141,143]. Maluleke et al. [64] observed a delay in the oxidation of Fe^2+^ ions in both non-adapted and Cu^2+^-adapted mixed mesophilic bacterial cultures (*L. ferriphilum, Ap. cupricumulans,* and *At. caldus*) in the presence of Sn^2+^ ions (1–10 g/L), but Cu^2+^-adapted cells exhibited better tolerance to tin ions than non-adapted cells (Figure 8). To address this problem, Ilyas et al. [110] employed a preliminary leaching stage using HNO_3_ to remove Sn-Pb solder before initiating the bioleaching process. Due to the complexity associated with the precipitation of tin compounds, only this limited number of studies has reported on the effects of tin and stannous ions on the bioleaching of PCBs.

Fungal bioleaching utilizes filamentous fungi due to their ability to produce organic acids (e.g., oxalic acid H_2_C_2_O_4_, citric acid H_8_C_6_O_7_, and gluconic acid H_12_C_6_O_7_) as metabolites that facilitate the solubilization of metal ions in solution [138]. *A. niger* and *P. simplicissimum* have been studied for tin leaching, demonstrating more promising results compared to bacterial leaching. This is attributed to their ability to bind metal ions into chelate complexes, thereby preventing salt hydrolysis and oxide precipitation. Brandl et al. [140] reported that both fungal strains could mobilize up to 65% of tin at a PCB concentration of 1 g/L. However, microbial growth was inhibited when the concentration of e-scrap exceeded 10 g/L. Preliminary studies using commercially available gluconic acid produced by *A. niger* showed that tin could be completely leached, even at PCB concentrations as high as 100 g/L.

Jadhav and Hocheng [142], in turn, developed a two-step process for the complete dissolution of metals from powdered PCBs. The process involved alkaline leaching with NaOH to remove the chemical coating, followed by bioleaching using the *A. niger* culture supernatant with the addition of 3.2% H_2_O_2_. This method achieved complete dissolution within 2 h at 80 °C. Pretreatment in NaOH and the addition of H_2_O_2_ revealed key factors for metal dissolution; otherwise, only 0.5–3.2% of tin could be extracted.

A low-energy method has been proposed by Alias et al. [146]. They used urban food waste (banana, orange, aubergine, courgette) and yard trimmings as substrates for the production of citric acid through solid-state fermentation by *A. niger*. The biological acid solution was able to extract metals from waste PCBs at a comparable level to commercial acid solutions. Notably, tin and iron were the most leached metals, even without the pre-treatment usually performed.

### 4.5. Solvometallurgical Treatment

Solvometallurgical methods developed for the treatment of PCBs, including the leaching of base and precious metals with ionic liquids [147] or deep eutectic solvents [148], are proposed as a promising alternative for environmentally friendly separation and recovery processes. While most research focuses on the extraction of copper, zinc, or even lead [147,148,149,150,151], the physical and chemical behavior of tin in contact with these novel solvents is reported only sporadically [104,152,153,154].

Early investigations [152,153] introduced novel technologies for solder removal from waste PCBs using ionic liquids. These studies employed 1-ethyl-3-methylimidazolium tetrafluoroborate [EMIM]BF_4_ [152] or 1-butyl-3-methylimidazolium tetrafluoroborate [BMIm]BF_4_ [153] as heating media for melting Sn-Pb solders (Table 2) from PCBs, enabling their dismantling within 10–12 min under stirring conditions. The selected temperature range of 240–250 °C was lower than the 270–280 °C typically required for standard PCB pyrolysis, thereby reducing the potential release of organic pollutants regardless of air availability. Despite the relatively high cost of ionic liquids, this method was recommended as a clean and non-polluting approach for recycling valuable materials from waste PCBs.

Recent studies [104,154] have highlighted aspects of the chemical behavior of tin and tin(IV) oxide in contact with deep eutectic solvents. Mishra et al. [104] compared the solubility of metal oxides (CuO, Fe_2_O_3_, ZnO, NiO, SnO_2_) in five systems: ethylene glycol-choline chloride, EG-ChCl; urea-choline chloride, Urea-ChCl; formic acid-choline chloride, FA-ChCl; oxalic acid-choline chloride, OA-ChCl; and malonic acid-choline chloride, MA-ChCl. In all cases (except FA-ChCl), ZnO exhibited the highest leaching ability and selectivity, while SnO_2_ showed low solubility, increasing in the following order (at 80 °C): EG-ChCl (0.02%) < MA-ChCl ~ Urea-ChCl (~0.1%) < FA-ChCl (1.1%) < OA-ChCl (10%). The solubility observed with OA-ChCl was comparable to that in an aqueous solution of oxalic acid (1 M), indicating high selectivity of this acid.

Similarly, Zhao et al. [154] investigated the behavior of metal oxides (CuO, Cu_2_O, Fe_2_O_3_, ZnO, PbO, SnO_2_) and silver (metal) in EG-ChCl, OA-ChCl, and glycolic acid-choline chloride GA-ChCl systems. While ZnO was consistently the most leachable material and silver resisted chemical action in all tested deep eutectic solvents, the solubility of SnO_2_ increased as follows (50 °C, 8 h): EG-ChCl (~2%) < GA-ChCl (20%) < OA-ChCl (70%). The high solubility of tin oxide in solvents containing oxalic acid, as well as in aqueous oxalic acid [104], can be attributed to the formation of soluble oxalate complexes Sn(C_2_O_4_)_3_^2−^ [154].

The leachability of metallic tin from PCBs was found to be significantly more effective [104], particularly at elevated temperatures or in the presence of an oxidizing agent (H_2_O_2_, potassium permanganate KMnO_4_). For instance, tin extraction from PCBs using FA-ChCl achieved up to 75% efficiency (100 °C, 21 h, thermally treated PCBs) or even 90% in the presence of H_2_O_2_ (100 °C, 24 h). The role of the oxidizing agent is further confirmed by the increased efficiency of tin leaching in the presence of KMnO_4_, reaching 42% (FA-ChCl, 80 °C, 21 h, pyrolyzed PCBs).

**Figure 9:**
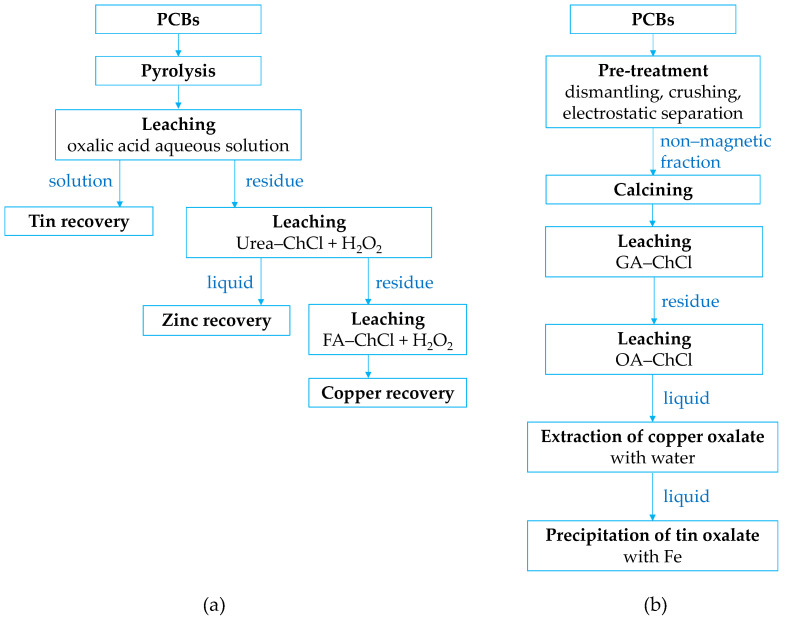
Schemes of metal recovery from waste PCBs using deep eutectic solvents: (**a**) based on ref. [104], (**b**) based on ref. [154].

Based on these findings, two alternative routes for tin recovery were proposed: (1) selective leaching of tin from pyrolyzed PCBs using an aqueous oxalic acid solution, followed by cementation, precipitation, or electrowinning (Figure 9a) [104]; or (2) recovery of tin oxalate as the final stage of PCB processing using a deep eutectic solvent based on choline chloride, preceded by physical separation pre-treatments (Figure 9b) [154]. 

## 5. Tin Recovery from LCD

### 5.1. Tin in Liquid Crystal Displays

Liquid crystal displays LCDs are widely used in various applications, including consumer electronics (e.g., flat-panel TVs, computer monitors, smartphones, tablets), digital displays, and control panels in medical instruments, industrial machinery, and gaming consoles, among others. The dominant and continuously increasing annual production of LCDs, estimated at 328.3 million m^2^ in 2024 [155], combined with their relatively short lifespans (ranging from 2 years for smartphones to 3–8 years for TVs [156,157]), generates a significant volume of waste on a global scale, necessitating efficient recycling [158].

The most valuable material in such waste is indium tin oxide ITO. In LCDs, ITO is present in two distinct layers; in active-matrix liquid-crystal displays, it serves as the common electrode in the color filter plane (on the front glass of the display), and in AM-LCDs, it functions as a pixel electrode in the thin-film transistor plane [159]. ITO is a transparent semiconducting material, typically applied as a coating with a thickness of 100–300 nm on LCD glass. It consists of a mixture of In_2_O_3_ (90–95%) and SnO_2_ (5–10%), with tin accounting for about 8% of its composition. This corresponds to average 0.1% tin in LCD units across various appliances (Table 5). In addition, indium (0.01–0.05%), aluminum (~0.1%), iron (~0.1%), chromium (~0.02%), nickel (~0.008%), titanium (~0.06%), zinc (~0.03%), strontium (~0.01%), arsenic (~0.015%), and mercury (~0.005%) can be found [160,161,162]. Schuster and Ebis [163] compared the compositions of the front and back sides of ITO glass (with the organic layer removed) and the LCD screen. The tin content differed between the two sides, with 9 ppm (92 ppm In) on the front side and 3.9 ppm (37 ppm In) on the back side of the ITO glass. Similarly, for the LCD screen, the tin content was 10 ppm (87 ppm In) on the front side and 7 ppm (29 ppm In) on the back side.

Kalmykova et al. [157] estimated that about 87.3 t of tin are embedded annually in TVs and monitors disposed of within the European Union, which corresponds to around 0.1% of the European tin market size (107,300 t in 2024 [164]). Although indium is the primary driver for LCD recycling, the recovery of coexisting tin presents greater challenges due to the similar chemical properties of the two metals, the lower tin content, and the high chemical resistance of tin(IV) oxide.

### 5.2. Physical Pre-Treatment

The first stage of metal recovery involves separating LCD panels from backlight components, circuit boards, cables, metallic parts, and polymer materials using an appropriate dismantling method. This can be performed manually (the most efficient and economical approach), mechanically, or, more rarely, through automation [165]. The resulting LCD panels have a sandwich structure comprising two types of glass substrates coated with an ITO film, with liquid crystal located between the glass layers and a polarizing film on the outermost surface [159]. To prepare the ITO glass for subsequent metal recovery, it is essential to remove the polarizing film and liquid crystal. This can be achieved through various methods [165]: (i) nitrogen or vacuum pyrolysis to convert combustible organic polymers into oil and gas [166]; (ii) a combination of physical and chemical processes, such as heating (thermal shock) to remove the polarizing film, crushing for size reduction, and acetone leaching to remove liquid crystal [167]; (iii) freezing in liquid nitrogen (thermal shock) to strip the polarizing film [168]; (iv) electrical disintegration using high currents to separate ITO glass without crushing [169]; or (v) conventional grinding [169,170]. In practice, methods such as cutting, crushing, and milling are commonly employed, as size reduction has a significant impact on the efficiency of subsequent hydrometallurgical processing.

Grinding ITO glass alters the material’s structure from crystalline to amorphous (particle size of 0.1 mm) [171], while simultaneously segregating metals into specific particle size fractions [170]. Rocchetti et al. [170] analyzed the granulometric distribution of ground LCDs (<10 mm), along with the concentrations of indium and tin. The distribution of metals across three material stocks was uneven. Tin was detected exclusively in one stock (260–0–0 ppm), while indium was present in all three stocks, albeit with a wide range of concentrations (53–130–110 ppm). The findings revealed that indium concentrations decreased with increasing particle size, ranging from 375 ppm for particles < 0.125 mm to 73 ppm for particles > 1.25 mm. In contrast, tin concentrations were significantly higher across the particle size fractions, varying from 484 ppm to 800 ppm in four fractions below 1.25 mm and decreasing to 254 ppm for particles larger than 1.25 mm. Notably, the highest tin content (800 ppm) was observed in the 0.125–0.25 mm particle size range.

### 5.3. Hydrometallurgical Treatment

Hydrometallurgical treatment of waste LCDs has been developed predominantly with a focus on indium recovery [165,167,168,169], while tin is typically regarded as being of secondary importance [170,171,172,173,174,175,176,177,178,179]. However, a comprehensive recycling process should also include the recovery of tin [174,176,178,180], despite its occurrence as a relatively chemically resistant oxide, its slow leaching kinetics, and the formation of stable Sn^4+^ ions in highly acidic environments.

Acid leaching is the preferred method for extracting metals from LCDs (Table 6). Sulfuric acid is the most commonly used lixiviant, capable of dissolving 50–70% of tin [169,172,173,175,176,178], with efficiency enhanced by ultrasound [173]. Process efficiency depends on acid concentration, with an optimal range of 2–9 M [172,173,175,176,178]; lower [172,175] or higher [173] concentrations reduce the leaching rate. The effect of temperature is inconsistent, with studies reporting both improved [176,178] and diminished [175] leaching. Similarly, leaching time can either enhance [172,173,176,178] or reduce efficiency [175]. The latter effect is evidenced by the secondary precipitation of SnO_2_ and elimination from acid sulfate solution 97% of stannic ions within 2 h at 70 °C (1 M H_2_SO_4_) [175] or over 95% within 48 h at room temperature (2 M H_2_SO_4_) [161].

The addition of H_2_O_2_, even at low concentrations, significantly inhibits tin leaching in H_2_SO_4_ solutions. For instance, Qin et al. [176] reported a decrease in the tin leaching rate from 86% to 52% when 1% H_2_O_2_ was added to 3 M H_2_SO_4_ at 85 °C. Similarly, the tin leaching rate was reduced when mixtures of H_2_SO_4_ with HCl or HNO_3_ in various proportions were employed.

Schuster and Ebis [163] compared the leachability of metals from the front and back sides of spent, untreated LCD screens using inorganic acids (nitric, sulfuric) and organic acids (maleic, glycolic). They confirmed the complete removal of tin from ITO glass with H_2_SO_4_. However, recovery efficiencies varied (48–100%) depending on whether the front or back side was subjected to treatment by remaining individual acids. In turn, oxalic acid proved ineffective for tin leaching (3.5%) from ITO glass (1655 ppm Sn) [181].

Although tin ions are much less prone to hydrolysis in HCl solutions [161], hydrochloric acid has rarely been used as an ITO leaching agent [161,171,179]. Yang et al. [179] investigated the effect of acid concentration (0.1–6 M) on tin leaching from crushed LCDs (20 ppm Sn) over 96 h. They observed that tin concentrations stabilized within 48 h, with the highest concentrations (8 mg/L) achieved at 1 M HCl. Additionally, they compared the leaching results in HCl with those obtained in HNO_3_, identifying 6 M HNO_3_ as more efficient, with a twofold increase in tin ion concentration (17 mg/L). Zhang et al. [171] reported that only trace amounts of tin were dissolved, even when ultrasonic leaching was applied. In contrast, Illés and Kekesi [161] recommended the use of HCl solutions due to the high indium recoveries (over 90%), although they did not discuss the behavior of tin during leaching. Instead, they proposed using ion exchange (anionic resin column) to separate indium ions through elution with HCl and recovering tin ions by elution with NaOH. In turn, Kim et al. [180] employed solvent extraction to separate tin from indium ions after leaching waste ITO in HCl. Nearly complete tin extraction was achieved using D2EHPA or PC88A extractants within 0.5 h from 1.4 M and 3.88 M HCl, respectively. In the subsequent step, 50% and 70% of tin could be stripped from the respective loaded organic phases of D2EHPA and PC88A. Pilot-scale experiments demonstrated successful selective tin extraction using the PC88A extractant within 10–15 min from 2 M HCl (O/A ratio of 3), followed by stripping with 12 M HCl.

**Figure 10:**
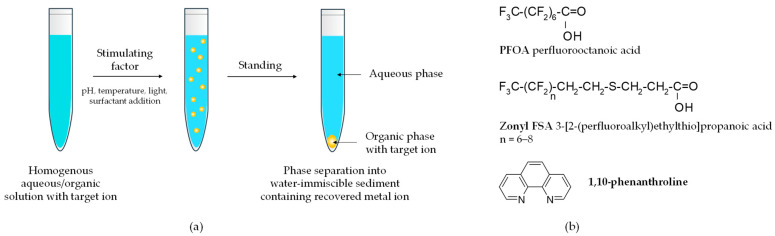
Scheme of HoLLE metal ion recovery (**a**) and applied compounds (**b**).

An advanced method for separating metal ions from acid chloride solutions was proposed by Kato et al. [182]. They used homogeneous liquid-liquid extraction HoLLE to achieve complete recycling of LCDs from mobile phones. HoLLE employs low-density organic solvents without the need for centrifugation. Unlike conventional solvent extraction, HoLLE begins with aqueous and organic phases in a homogeneous state during the extraction of target ions, eliminating the need for intensive mechanical mixing to increase phase interface contact (Figure 10).

In laboratory practice, aqueous solutions containing metal chelates (e.g., 1,10-phenanthroline) are used. Phase separation into a water-immiscible sediment containing the target metal ions (chelates) is triggered by changes in pH, temperature, light, or the addition of organic compounds such as surfactants (e.g., PFOA, Zonyl FSA) or water-miscible organic solvents (e.g., acetone). This process enables a 100- to 100,000-fold concentration of metal ions into a microvolume within minutes. Although the research [182] primarily focused on indium separation, it demonstrated that over 90% of tin ions from a simulated acid chloride leachate could also be recovered into the sedimented liquid phase using the HoLLE method with 1,10-phenanthroline, Zonyl FSA, and acetone as the separating system. Unfortunately, this HoLLE process was selective only towards calcium and strontium ions, transferring, in addition to indium and tin species, iron and aluminum ions into the sedimented organic phase.

Recently, novel adsorbents for the recovery of tin(IV) ions from acidic chloride, nitrate, or sulfate solutions have been developed [183,184]. Qin et al. [183] prepared a macroporous silica SiO_2_ adsorbent embedded with a styrene-divinylbenzene polymer P and impregnated with the D2EHPA extractant. The D2EHPA/SiO_2_-P adsorbent demonstrated rapid adsorption (10 min) and remarkable selectivity for stannic ions (100% efficiency) over other metal ions (adsorption efficiency below 5%) in a 6 M H_2_SO_4_ solution derived from leaching of LCDs. The high performance of the adsorbent (evaluated in batch experiments) was attributed to the action of P=O and P-O bonds during the monolayer chemical adsorption of Sn^4+^ ions. Complete desorption was achieved by breaking Sn-O bonds in 0.5–3 M NaOH. Similarly, Wang et al. [184] synthesized a silica-based adsorbent loaded with the P507 extractant (containing nitrogen and phosphorus donors) for industrial separation and recovery of stannic ions. This adsorbent was fabricated by the in situ growth of a covalent organic framework COF on a silica substrate combined with vacuum impregnation. The P507@COF-TpAzo/SiO_2_ adsorbent achieved nearly 100% tin(IV) removal (in both batch and column adsorption) from HCl (60 mg Sn^4+^/g) and HNO_3_ (92 mg Sn^4+^/g) solutions, leaving other metal ions in the aqueous phase. Complete elution of tin ions was accomplished using NaOH solutions, preceded by the removal of impurity metal ions through rinsing with ultrapure water.

Leaching of ITO from waste LCDs generates solutions containing low concentrations of metal ions, typically ranging from few [172,179] to several hundred mg/L [161,175]. Consequently, the methods based on ion exchange, extraction or adsorption, as described above, are most often considered the most suitable for separating and concentrating ions in such solutions. Traditional electrowinning methods are not applicable in these cases. However, Grimes et al. [185] developed a three-step electrolysis method that allows for the selective separation and recovery of lead, tin, and indium from acidic nitrate solutions containing 50 mg/L of each metal ion. This method employed a specially designed system with cylindrical mesh electrodes, where the cathode is positioned between two anodes. Selective recovery was controlled by adjusting the electrolyte composition: (i) in the first step, 97% of lead was selectively recovered using 0.1 M HNO_3_; (ii) in the second step, 94% of tin was recovered from the lead-depleted electrolyte after adding SCN^−^ ions; and (iii) in the third step, 98% of indium was recovered on the mesh anode as indium(III) oxyhydroxide.

Alternatively to LCD leaching with strong inorganic acids, unconventional methods using complexing agents have also been explored. Yáñez-López et al. [174] developed an ecofriendly process for metal separation using sodium citrate H_5_C_6_O_7_Na_3_ or citric acid H_8_C_6_O_7_. To enhance the solubilization of tin oxide, they employed hydrazine N_2_H4 to reduce tin(IV) to tin(II), which forms complexes with citrate ions:

SnO_2_ + N_2_H_4_ →  SnO + 2H_2_O + N_2_ (16)

SnO + H_5_C_6_O_3_^3−^ + 2H^+^ →  [Sn(H_5_C_6_O_7_)]^−^ + H_2_O (17)

The leaching was carried out at a pH of 5, adjusted with H_2_SO_4_ or HNO_3_ in 1 M H_5_C_6_O_7_Na_3_, and with NaOH in 1 M H_8_C_6_O_7_. Depending on the adjusting compounds, they observed a continuous increase in tin recovery over time (12 h), reaching up to 40% after 12 h for H_2_SO_4_, a maximum of 40% after 3 h for HNO_3_, and a maximum of 95% after 6 h for NaOH. The presence of maxima on the kinetic curves, followed by a decrease in tin recovery, was attributed to a secondary cementation reaction by other less noble metals, such as metallic iron or aluminum, present in the LCD glass structure. To address this issue, H_2_O_2_ was added, but it did not change the behavior of tin. Therefore, a pretreatment of the LCD powder to remove 60% of iron with H_2_SO_4_ was introduced, followed by leaching with a mixture of H_8_C_6_O_7_ and N_2_H_4_ (pH 5, NaOH). In this process, about 20% of tin was recovered in the first stage (0.7 h), followed by 65% tin recovery in the second stage (19 h). Interestingly, during the second stage, three distinct changes in tin concentration in the solution were observed: an increase up to 6 h, followed by a plateau until 15 h, and then a further increase in recovery until the end of the process (19 h). Meanwhile, indium recovery stabilized at 95% after 7 h. Notably, tin recovery was enhanced by increasing the citrate concentration, but an inhibitory effect was observed due to iron ions, which also used citrate for complex formation. To mitigate this effect, the addition of sodium phosphate was tested to keep iron in a form other than a citrate complex, but this proved ineffective.

In turn, Toache-Pérez et al. [177] reported a novel method for the recovery of metals (indium, erbium, gadolinium) from LCDs using ultrasound-assisted leaching in a mixture of sodium pyrophosphate Na_4_P_2_O_7_ and H_2_O_2_, followed by magnetic separation to recover the metals in metallic form:

SnO_2_ + 2H_2_P_2_O_7_^2−^ →  SnP_2_O_7_ + 3H_2_O + + 2H^+^ (18)

SnP_2_O_7_ + 2H_2_O_2_ →  Sn + 2H^+^ + 3O_2_ + H_2_P_2_O_7_^2−^ (19)

Tin leaching showed high selectivity over indium in the presence of ultrasounds, although tin recovery was relatively low (~23%). The leachability of tin from the LCD was low and more dependent on time than pH; however, these parameters enhanced the dissolution of erbium and iron. The addition of H_2_O_2_ reduced the metal ions to their elemental forms, which could be recovered magnetically as a mixture of tin with indium, erbium, and gadolinium, although their phase composition was not experimentally confirmed.

### 5.4. Biohydrometallurgical Treatment

The application of biohydrometallurgical processing of waste LCD panels has received increased interest due to the possibility of ecologically recovering valuable metals. However, most research studies focus on the use of bacteria or fungi for the recovery of indium, neglecting the assessment of tin recovery potential [186,187,188,189,190] and only a few studies have shown the behavior of this metal during bioleaching with acidophile bacteria, but not fungi (Table 7).

Willner et al. [191] used an adapted mixed bacterial culture (*At. ferrooxidans* and *At. thiooxidans*) for comparative studies of tin leachability from powdered LCDs (1000 ppm In, 250 ppm Sn) using different media, i.e., 9 K and H_2_SO_4_, both with elemental sulfur addition (control tests under sterile conditions were conducted in parallel). In the 9 K medium, the extraction rate gradually increased to 90% within 14–21 days, followed by a decline, which was more pronounced in the presence of bacteria. In the second case, the solution pH remained stable over time at about 1.8, whereas in the absence of microorganisms, it gradually increased, reaching up to 3 after 35 days. In contrast, bioleaching in the H_2_SO_4_ medium remained below 10% and decreased after the 10th day in the acid control sample. The higher tin leaching rates observed in the 9 K medium were attributed to the intensified bacterial production of Fe^3+^ ions, which act as an oxidizing agent, although the mechanism of oxide dissolution was not interpreted. Further studies [192] also investigated the effect of specific pure bacterial cultures on tin bioleachability at different pulp densities (L/S 100 or 50). It was shown that lower pulp density resulted in higher indium and tin dissolution. The *At. ferrooxidans* bioleaching system demonstrated over 30% higher metal extraction within a shorter time compared to *At. thiooxidans*, indicating the special role of iron ions and *At. ferrooxidans* in tin recovery. Unfortunately, none of the publications identified the final products of tin bioleaching (only jarosite was identified [191]), though its concentration at the end of the 35-day period with the mixed bacterial culture was about 3 mg/L in the 9 K medium and about 0.7 mg/L in the H_2_SO_4_ medium (practically the same as after one day of bioleaching). Notably, tin leaching efficiency was higher than that of indium in the presence of *At. ferrooxidans* during shorter process durations (up to 15 days) [191,192]. Prolonged bioleaching (35 days) improved indium leachability, reaching or exceeding tin leaching efficiency, probably due to the absence of secondary precipitation of indium compounds.

### 5.5. Solvometallurgical Treatment

Solvometallurgical approaches for the recovery of metals from ITO glass are uncommon. Jin et al. [193] used three deep eutectic solvents based on choline chloride ChCl and carboxylic acids (oxalic OA, malonic MA, and succinic SA acids) for leaching ITO powder. They observed a somewhat preferred leaching of tin over indium and an increasing tin leaching rate (L/S 40, 80 °C, 5 h) in the following order: SA-ChCl (71%) < MA-ChCl (95%) < OA-ChCl (~100%). Analysis of the leaching solution revealed that SnO_2_ from ITO was transformed into soluble Sn(IV) species, such as [Sn(OH)_3_H_2_O]^+^ and [Sn(MA)Cl_4_]^2−^ ions. The tin-containing species in the leaching solution were then completely precipitated as cassiterite SnO_2_ through water dilution and hydrothermal treatment (120–180 °C):

[Sn(OH)_3_H_2_O]^+^ + OH^−^ →  SnO_2_ + 3H_2_O (20)

The final tin product was contaminated with indium compounds (Sn/In ratio~3), but the remaining liquid could be used for the recovery of In_2_O_3_ by precipitation with ammonia, followed by calcination.

Other studies [194,195] employed ionic liquids for the separation of tin and indium from acidic aqueous solutions generated during LCD leaching. Deferm et al. [194] used Cyphos*^®^* IL 101 (tri(hexyl)tetradecylphosphonium chloride) and Aliquat*^®^* 336 (trioctylmethylammonium chloride) for the selective recovery of indium (over 95%) from acid chloride solutions (0.5–12 M HCl) during solvent extraction. The experiments showed that although the distribution coefficient for Sn(IV) ions (over 5000) was much higher than that for In(III) (up to 690), high In(III)/Sn(IV) separation factors (over 9.8 for Cyphos*^®^* IL 101 and over 162 for Aliquat*^®^* 336, both in 0.5 M HCl) suggested easier indium stripping, though this was not verified experimentally. In turn, Dhiman and Gupta [195] used Cyphos*^®^* IL 104 (tetradecyl-(trihexyl)phosphonium bis-(2,4,4-trimethylpentyl) phosphinate) in a similar system. Although they also focused on indium recovery, Sn(IV) ions were more prone to extraction in 1 M HCl (90% Sn vs. 70% In), while for more concentrated acid (5–7 M), no selectivity was observed. Similarly, varying the concentration of extractant in the range of 0.1–1 M did not affect the selectivity of indium-tin separation. After complete stripping of the loaded organic phase with HNO_3_ or HCl solutions, both metals could be separated by selective precipitation of indium as oxide, with tin as sulfide.

## 6. Conclusions

**Figure 11:**
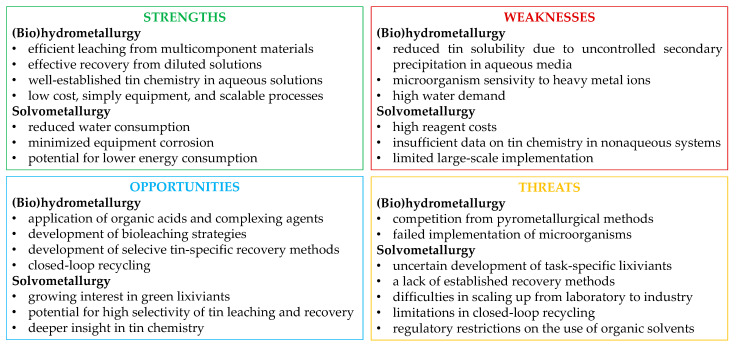
SWOT analysis of tin recovery from low-grade secondary sources.

The recovery of tin from obsolete printed circuit boards and liquid crystal displays has garnered significant attention due to the increasing demand for electronic waste recycling; however, the projected limited availability of the metal in the future should serve as a clear signal to prioritize tin recovery even from such low-grade sources. Both waste materials are multicomponent and contain low concentrations of tin (few percentages in PCBs and ppm in LCDs) in different chemical forms (metal or alloy in PCBs, SnO_2_ in LCDs). As a result, (bio)hydrometallurgical and solvometallurgical methods have emerged as suitable and sustainable techniques for the extraction and separation of tin, although the choice of the most efficient extraction method largely depends on the material composition, process scalability, economic viability, and potential environmental impact (Figure 11).

Hydrometallurgical methods for tin recovery from PCBs were significantly developed, driven by the need to remove tin-based solders and enhance the dismantling of PCB components. This led to the development of several selective recovery methods that utilize both physical properties (such as melting) and chemical reactions (dissolution). Leaching in strong inorganic acids under optimal conditions can dissolve over 99% of tin, but separation issues arise due to hydrolytic secondary precipitation of SnO_2_, which is problematic when using HNO_3_ or H_2_SO_4_. Therefore, an optimal approach seems to involve using HCl-based solutions or unconventional organic acids such as methanesulfonic or oxalic acids. Bioleaching of the waste is generally ineffective for tin leaching due to the precipitation of secondary oxides. This represents a challenge for further research, particularly in adapting bacteria or fungi to leach in solutions containing heavy metal ions, which are toxic to microorganisms. Additionally, a deeper understanding of the behavior of tin species during bioleaching is required. The use of deep eutectic solvents based on choline chloride and carboxylic acids, particularly in oxidizing environments, still requires development to optimize conditions. Although this approach is more expensive than traditional hydrometallurgical methods, it could facilitate selective tin recovery.

Waste LCDs contain traces of tin(IV) oxide, which is more resistant to chemical action than metallic tin. As a result, selective recovery of tin from these materials is highly challenging. The application of classical inorganic acids such as H_2_SO_4_ or HCl shows moderate extraction efficiency, but good prospects exist for using unconventional leachants that form soluble chelate complexes with tin ions, offering environmentally friendly alternatives. In contrast to bioleaching of PCBs, the use of microorganisms for ITO glass leaching is scarce, which suggests potential for development, especially that the effectiveness of biological leaching SnO_2_ appears to be higher than that of metallic tin from solders over shorter periods. Since the resulting leachates are diluted, there is a need to develop advanced methods for selective separation and preconcentration of tin ions before final product recovery. Methods based on ionic properties, such as ion exchange with liquids or resins, or selective adsorbents, are generally recommended. Finally, solvometallurgical methods should also be developed, as they appear to be effective. Although they are not currently cost-effective due to the toxicity of the solvents, which may limit their sustainability in the long run, they could prove efficient in selectively recovering valuable accompanying metals in the future.

All methods face significant challenges, including the need to increase metal recovery rates, selectivity, and minimize the generation of secondary products. Future research should focus on developing hybrid approaches that combine the strengths of (bio)hydrometallurgical and solvometallurgical techniques to optimize economically viable recovery of tin from low-grade electronic waste.

## Figures and Tables

**Table 1:** Tin contents (in wt%) in waste printed circuit boards [43,47,48,50,54,61,62,63,64,65,66].

Routers	Computers	Mobile Phones, Smartphones	TV Board	Copy Machine	Fax Machine	Printer	Central Processing Unit
1.3–9.3	0.7–10.3	0.02–4.7	1.4–6.4	2.5	3.0	1.0	1.8

**Table 2:** Key properties of tin and tin-based alloys related to PCBs physical pre-treatment [73,74].

Material *	Melting Point, °C	Density, g/cm^3^	Electrical Resistivity, μΩ∙m	Thermal Conductivity, W/m∙K	Brinell Hardness, HB
Sn metal	232	7.3	0.12	73	4
Sn-37Pb alloy	183	8.4	0.25	50	17
Sn-Ag alloys	~221	7.4	0.12	55	15
Sn-Cu alloys	~227	7.3	0.12	66	9
Sn-Ag-Cu alloys	~217	7.4	0.13	58–62	15
Sn-Bi alloys	138	8.1	0.35	30	24

* Mass magnetic susceptibility of tin and its alloys is on the order of 10^−9^ m^3^/kg.

**Table 3:** Tin leaching and recovery from waste PCBs at optimal conditions.

Tin Content in PCBs, %	Leaching Conditions	Leaching Rate, %	Tin Recovery Conditions	Final Product	Recovery Rate, %	Ref.
Acid Leaching						
3.3	L/S 5, 2 M HCl, 80 °C, 6 h	91.8	-	-	-	[89]
?	L/S 20, 4.5 M HCl, 90 °C, 1 h	97.8	Precipitation with NaOH at pH 1.9	Na_2_SnO_3_	?	[90]
2.6	L/S 10, 2 M HCl, 75 °C, 1 h	88.0	Cementation with Al	Sn-Pb	97.0	[91,92]
3.1	L/S 10, 3 M HCl, 60 °C, 2 h	89.1	Precipitation with NaOH under pH control	?	98.2	[93]
	L/S 10, 3 M HCl + 1 M HNO_3_, 60 °C, 2 h	98.1		SnO, SnO_2_	85.8	
	L/S 10, 3 M HCl + 1 M H_2_SO_4_, 60 °C, 2 h	90.5			96.3	
2.5	L/S 12.5, 4.9 M HCl, 74 °C, 3 h	97.6	Precipitation with NaOH at pH 3; calcination	SnO_2_	99.9	[94]
0.1	L/S 3, 1–6 M HNO_3_, 80 °C, 6 h	<1%	SnO_2_·H_2_O dissolution in 1.5 M HCl; electrowinning	Sn	100	[95]
4.6	L/S 20, 1 M H_2_SO_4_, 55 °C, 1.5 h	26.7	-	-	-	[96]
	L/S 10, 1 M H_2_SO_4_ + CuSO_4_ (n_Cu_/n_Sn_ 1.6), 65 °C, 1.5 h	95.2	Hydrolysis precipitation: 10% H_2_O_2_, 80 °C, pH 3, 2 h	SnO_2_	92.7	
16.2 *	L/S 20, 2 M H_2_SO_4_, 80 °C, 3 h	~20	-	-	-	[97]
2.5	L/S 10, 2 M H_2_SO_4_, 80 °C, 8 h	100	-	-	-	[98]
12.7 *	L/S 3.3, 6 M HCl + 3 M NaCl, 25 °C, 24 h	94.8	Precipitation with NaOH at pH 3	amorphous	97.4	[99]
6.2 *	L/S 45, 5 M HCl + Cl_2_ (anode, at 4 A), 30 °C, 1 h	99.4	-	-	-	[100]
2.2	L/S 13, 1 M HCl + 0.08 M SnCl_4_, 50 °C, 4 h	93.5	-	-	-	[101]
3.8	L/S 20, 2 M HCl + 1.17 M NaBr + 0.77 M Br_2_, 23 °C, 10 h	96.8	-	-	-	[50]
	L/S 20, 1.2 M HNO_3_ + 1.17 M NaBr + 0.77 M Br_2_, 23 °C, 10 h	97.1	-	-	-	
	L/S 20, 2.7 M H_2_SO_4_ + 1.17 M NaBr + 0.77 M Br_2_, 23 °C, 10 h	99.2	-	-	-	
?	3 M HBF_4_, 0.4 M H_2_O_2_, 20 °C, 0.5 h	98.5	-	-	-	[102]
?	3.5 M CH_4_SO_3_, 0.5 M H_2_O_2_, 20 °C, 0.75 h	99.2	-	-	-	[103]
7.9 **	L/S 20, 1 M H_2_C_2_O_4_, 80 °C, 1 h	92.3	-	-	-	[104]
**Alkaline Leaching**						
3.3	L/S 5, 1 M NaOH, 90 °C, 2 h	62.4	-	-	-	[89]
8.6	L/S 4, 2.5 M NaOH, p_O2_ 2 MPa, 150 °C, 3 h	98.2	Precipitation of PbS and ZnS; electrowinning	Sn	86.2	[105]
**Chelating Leaching**						
16.2 *	L/S 30, 0.1 M Na_2_-EDTA, pH 5, 80 °C, 3 h	100	Precipitation with NaOH at pH 9, 60 °C	SnO_2_ nanoparticles	?	[97]
**Electrochemical dissolution of PCB Anode**						
13.8	Sn^2+^-CH_4_SO_3_, 40 °C, 2 A/dm^2^	85	Simultaneous cathodic deposition	Sn	?	[106]
2.6	3 M NaOH, 80 °C, 3 A/dm^2^, 2 h	100		Sn	?	[107]

L/S—liquid-to-solid ratio. -—not investigated. ?—no data. * Metallic fraction after physical separation. ** In pyrolyzed PCBs (1.9% Sn).

**Table 4:** Tin bioleaching from waste PCBs.

Microorganism	Leaching Agents	Bioleaching Conditions	Tin Content in PCBs, %	Leaching Efficiency, %	Ref.
Bacteria Leaching					
*Acidithiobacillus ferrooxidans*	Fe^3+^, H_2_SO_4_	L/S 1000, pH 2.6, 30 °C, 10 days	2.3	0 *	[140]
*Acidithiobacillus thiooxidans*	H_2_SO_4_				
*Acidithiobacillus ferrooxidans*	Fe^3+^, H_2_SO_4_	L/S 285, pH 1.8, 30 °C, 4 days	3.3	0 **	[144]
*Leptospirillum ferriphilum*	Fe^3+^	L/S 1000, 38–150 μm, pH 1.8, 30 °C, 2 days	18	21	[145]
*Acidithiobacillus ferrooxidans*	Fe^3+^, H_2_SO_4_				
*Acidithiobacillus caldus*	H_2_SO_4_				
*Leptospirillum ferriphilum*	Fe^3+^	L/S 1000, pH 1.4, 37 °C, 3 h	0.1–3 g/L	0 *	[64]
*Acidiplasma cupricumulans*	Fe^3+^, H_2_SO_4_				
*Acidithiobacillus caldus*	H_2_SO_4_				
**Fungal Leaching**					
*Aspergillus niger*	H_2_C_2_O_4_, H_8_C_6_O_7_, H_12_C_6_O_7_	L/S 1000, pH 3.0–3.5, 30 °C, 21 days	2.3	38	[140]
*Penicillium simplicissimum*				65	
*Aspergillus niger*		L/S 500, 30 °C, 1 day	0.98	1.5	[142]

* Secondary SnO precipitation in solution. **—No dissolved Sn or ions entrapped in jarosite.

**Table 5:** Tin contents (in ppm) in LCD panels * [160,161,162].

Computer Monitor	TV Monitor	Smartphone	iPhone	Mobile Phones	Tablet	Notebook	Laptop
10–46	18 ± 7	3 ± 2	1276	15–4470	16 ± 10	11 ± 4	110

* Based on tin mass (in g) in 1 g LCD, 1 ppm = 10^−4^%.

**Table 6:** Tin leaching and recovery from waste LCDs at optimal conditions.

Tin Content in LCDs, ppm	Leaching Conditions	Leaching Rate, %	Tin Recovery Conditions	Final Product	Recovery Rate, %	Ref.
Acid Leaching						
260 ± 30	L/S 5, 2 M H_2_SO_4_, 80 °C, 1 h	60	-	-	-	[170]
100 ± 20	L/S 10, 2 M H_2_SO_4_, 70 °C, 8 h	60	-			[172]
? *	L/S ?, 18 M H_2_SO_4_, 60 °C, 0.5 h	50	-	-	-	[173]
	L/S ?, 18 M H_2_SO_4_, 60 °C, 0.5 h, us	70				
	L/S ?, 9 M H_2_SO_4_, 60 °C, 0.5 h, us	100				
12,800 **	L/S 8, 4 M H_2_SO_4_, 70 °C, 2 h	80	-	-	-	[175]
	L/S 8, 1 M H_2_SO_4_, 70 °C, 2 h	3	hydrolytic precipitation during leaching	SnO_2_	97	
100	L/S 6, 3 M H_2_SO_4_, 85 °C, 1 h	86	-	-	-	[176,178]
4000 *	L/S 10, 0.8 M HCl, 25 °C, 1 h, us	31	-	-	-	[171]
**Chelating Leaching**						
1392	L/S 50, 1 M H_5_C_6_O_7_Na_3_, 0.2 M N_2_H_4_, pH 5 (H_2_SO_4_), 25 °C, 12 h	40	-	-	-	[174]
	L/S 50, 1 M H_5_C_6_O_7_Na_3_, 0.2 M N_2_H_4_, pH 5 (HNO_3_), 25 °C, 3 h	40				
	L/S 50, 1 M H_5_C_6_O_7_Na_3_, 1.5 M H_2_O_2_, pH 5 (HNO_3_), 25 °C, 3 h	40				
	L/S 50, 0.5 M H_8_C_6_O_7_, 0.2 M N_2_H_4_, pH 5 (NaOH), 25 °C, 3 h	95				
835	L/S 50, 0.05 M Na_4_P_2_O_7_, 3% H_2_O_2_, pH 3, 25 °C, 2 h, us	1	magnetic separation from solid residue	Sn	72	[177]
	L/S 50, 0.05 M Na_4_P_2_O_7_, 3% H_2_O_2_, pH 6, 25 °C, 1 h, us	23	-	-	-	

L/S—liquid-to-solid ratio; us—ultrasonic leaching; -—not investigated; ?—no data. * Non-crushed LCDs (otherwise powdered). ** Pyrolyzed LCD.

**Table 7:** Tin bioleaching from waste LCDs at optimal conditions.

Microorganism	Leaching Agents	Bioleaching Conditions *	Tin Content in PCBs, ppm	Leaching Efficiency, %	Ref.
*Acidithiobacillus ferrooxidans*	Fe^3+^, H_2_SO_4_	L/S 100, 9 K, 30 °C, 14 days	250	90	[191]
*Acidithiobacillus thiooxidans*		L/S 100, 9 K, H_2_SO_4_, 30 °C, 21 days		10	
*Acidithiobacillus ferrooxidans*	Fe^3+^, H_2_SO_4_	L/S 100, 9 K, 30 °C, 15 days		98	[192]
*Acidithiobacillus thiooxidans*	H_2_SO_4_	L/S 100, WJ, 30 °C, 35 days		65	
*Acidithiobacillus ferrooxidans*	Fe^3+^, H_2_SO_4_	L/S 100, 9 K, 30 °C, 15 days		98	
*Acidithiobacillus thiooxidans*					

* 9 K—(NH_4_)_2_SO_4_, KCl, MgSO_4_, KH_2_PO_4_, FeSO_4_, S, H_2_O—Silverman-Lundgren medium; WJ—(NH_4_)_2_SO_4_, CaCl_2_, MgSO_4_, K_2_HPO_4_, FeSO_4_, S, H_2_O—Waksman and Joffe medium.

## Data Availability

No new data were created.
